# On Quantity and Quality in Human Knowledge

**DOI:** 10.1007/s13752-015-0218-y

**Published:** 2015-07-30

**Authors:** Jorge Wagensberg

**Affiliations:** Faculty of Physics, University of Barcelona, Barcelona, Spain

**Keywords:** Art, Complexity, Dialectics, Entropy of knowledge, Intelligibility, Interdisciplinarity, Objectivity, Quality of knowledge, Revelation, Science

## Abstract

Any discipline of human knowledge is characterized by three fundamental elements: the complexity of its content, the method used for its elaboration, and the language used for its expression. This article argues that any method for making knowledge is a particular combination of three main components that we can call (a) science, (b) art, and (c) revelation. The right combination depends on the complexity of the slice of reality that we wish to understand in each case. Is there a relationship between the quantity and quality of a particular piece of knowledge and the quantity and quality of its eventual audience? Such a relationship serves, I believe, to avoid certain old misunderstandings.

## Introduction

There is, one might say, an unwritten conservation law that combines the concepts of quantity and quality in relation to human knowledge: the greater the quantity, the lower the quality. Before we go on to define these supposedly conflicting concepts with a degree of rigor, there are a number of observations to be made that seem beyond doubt. For example, Mozart, Bach, Beethoven, and Stravinsky have never been top of the record charts; the lists of best-selling books are crammed with self-help books, as the idea of the best seller in no way resembles a book of poems by Leopardi; and cultural producers and agents choose stimuli more on the basis of the level of consumption rather than their intrinsic value. It is as if quantity as a concept were opposed to that of quality, or at least not entirely compatible with it. It may also be that this is all the consequence of a colossal and ancient misunderstanding.

In this article, we will take as our starting point the language of mathematics to offer two definitions of the concepts of quantity and quality in relation to knowledge. To do this, we will base ourselves on two fundamental ideas: classes of equivalence and classes of order. We will then go on to propose a classification of any class of knowledge according to two basic criteria: one based on the method for obtaining it and the other based on the language used. This approach invites us to distinguish between two classes of universality of every type of knowledge: the universality of its viability as an object (the universality of its content) and the universality of its viability as a subject (the universality of its audience).

## Classes of Order and Classes of Equivalence

The different families of numbers—natural numbers, integers, rational, real, complex, and other number sets—were invented to evaluate or measure quantities. Natural numbers (1, 2, 3, and so on) count and order while making the quality of the concepts abstract. A shepherd who only owns three sheep has no need of abstraction in order to know if one is missing when the sun comes up. He knows them all and he identifies them all by name–Teresa, Jane, and Dolly, for example. He does not even need to know that there are three of them. But if he has 648, then it is better for him to count them and know how many he has. He does not even need to know what they are called or what they look like. What do a sheep, a black sheep, a white sheep, a cloud, a tree, a planet, or a depression have in common? The number one! Natural numbers count and order with abstraction other qualities that can be remembered. And so sets of numbers are enlarged to include the set of integers (…−3, −2, −1, 0, 1, 2, 3,…) on either side of a reference point (zero) but which do not divide or distribute; rational numbers (…–1/2, 1/3, 1/8, 22/7,…), which do divide and distribute but which do not serve to calculate the quotient between the perimeter or the diameter of a circumference or the square root of two; real numbers which also calculate geometrical or algebraic ratios (such as π or $\surd 2$) but which cannot calculate everything such as the square root of a negative number; and complex numbers, which can extend even more the solution of many problems.

In fact, natural numbers are all that is required to measure and order quantities (to measure in general any real magnitude). Strictly speaking, we can term anything that can be represented by a natural number a “quantity.” This occurs when a collection of objects (a, b, c, and so on) admits a relation R between them that fulfills three simple properties, the *reflexive*, the *transitive* and the *antisymmetric*:

1 $$\begin{aligned} &{\text{aRa}} \,\quad\qquad\qquad\qquad Reflexive \\ &{\text{aRb}},{\text{ bRc}} \Rightarrow {\text{aRc}} \quad\,\,\, \,Transitive \\ &{\text{aRb}},{\text{ bRa}} \Rightarrow a\, = a \quad Antisymmetric \\ \end{aligned}$$

for any a, b, c in the group (Brown 2006).

This relation, the relation of order, is what makes one of the important operations of human knowledge possible: comparing. Two objects related by an order relation R represent quantities that can be compared with each other (a is smaller than b, a is larger than b, or a is equal to b).

When we talk of qualities, it is curious that we tend to do the same thing; in other words, we also have the need to compare (this has a higher, lower, or equal quality as). How does one define the quantity of a quality? What is clear is that classes of order are not sufficient to compare qualities. The definition of quality requires the prior definition of the class concept and of the so-called classes of equivalence. An R relation in a collection of objects (a, b, c, etc.) defines a class of equivalence if it fulfills three properties, the *reflexive*, the *transitive* and the *symmetric*:

2 $$\begin{aligned} &{\text{aRa}}\qquad \qquad \qquad\quad Reflexive \hfill \\ &{\text{aRb}},{\text{ bRc}} \Rightarrow {\text{aRc}}\quad\,\,\, Transitive \hfill \\ &{\text{aRb}} \Rightarrow {\text{bRa}}\qquad \quad \,\,\,\,Symmetric \hfill \\ \end{aligned}$$

whatever the three a, b, c elements in the collection are (Brown 2006). In other words: two objects are of the same class of equivalence (or are equivalent or, if you like, are comparable) if there is an equivalence relation R between them. The most important consequence of a division of a collection into classes of equivalence lies in the fact that every object belongs to a class, and there is no object that belongs to two classes at the same time. This is the perfect classification, like Mendeleev’s periodic table or Linnaeus’ classification of species of living things. This mathematical structure makes the first relation (with a certain, not trivial sense) between quantity and quality possible. Let us suppose that a collection of *n* objects makes it possible to define two relations, one of order and the other of equivalence. In this case, we have the possibility of a double comparison: one of quantities between the objects thanks to the relation of order; and the other a comparison between the elements in a single class thanks to the relation of equivalence. The relation of order introduces a criterion of quantity (weights, for example: objects of equal weight belong to the same class of order; or age, for example: elements of equal age belong to the same class of order) so that each class of equivalence, defined with the same set of elements, has also an assigned quantity: the sum of the quantities of the elements that belong to it (i.e., the sum of weights or of ages). In other words, a classification of order permits degrees in the comparison between objects according to one criterion. In a classification of equivalence, the comparison between two objects is limited to determining whether they are or are not of the same class.

In any event, the distribution of quantities by classes has a measure, a measure that combines quantities and qualities. This is the well-known Shannon entropy, which measures the diversity of occupation of a classification, be it of order or equivalence, according to the distribution of a collection of objects in classes (Shannon 1948):

3 $$S\, = \, - \mathop \sum \limits_{i = 1}^{n} p(i)\, lg_{2}\, p\left( i \right)\,\quad {\text{bits}}/{\text{object}}$$

where *p*(*i*) = *n*(*i*)*/N* and where *n*(*i*) is the quantity of objects of quality *i* and where $N = \mathop \sum \nolimits_{i = 1}^{n} n(i)$ is the total quantity of objects in the collection and *n* the total number of different classes. Shannon entropy is a number between 0 and the *lg*_2_*n* that measures how the quantity is distributed among the quality. The lower limit corresponds to minimum diversity (all the objects in the same class) and the upper limit to a uniform distribution of all the objects among all the possible qualities.

In mathematical information theory in general and in statistical mechanics in particular, entropy is used to calculate the most probable structure of a system with all the information available in each case. The idea is to maximize entropy in known conditions. This is known as MaxEnt (maximum entropy formalism (Jaynes 2003)) and is a generalization of the famous second law of thermodynamics. It is a powerful and universal method that enables us to make the best possible prediction using all the information available in each case. Using this method, strong parallels have been found between questions as disparate as statistical mechanics (Jaynes 1957), marine ecosystems (Lurié and Wagensberg 1983), fractal structures in mathematics (Pastor-Satorras and Wagensberg 1998), and Zipf’s law in linguistics (Mandelbrot 1965). It is, then, the basis of what we could call a theory of complexity according to which similar behaviors can be deduced in systems that bear no relation to each other except in one thing: they are complex systems. Accordingly, we can refer to entropy as quantitative measure of quality. We will now go on to apply these ideas to the notion of the quantity and quality of human knowledge. Firstly, however, we will briefly attempt to define what it is that we term *human knowledge*.

## The Entropy of Knowledge

Knowledge is packaged thinking capable of leaving one mind and reaching another one after traversing the reality of the world. The thought that cannot be communicated from one mind to another will never be knowledge. The thought may in principle be presumably infinite (an undefined number of nuances are needed to determine it); knowledge, however, is necessarily finite to enable it, duly packaged, to traverse reality and reach another mind, where it is decoded. A poem, a gesture, a musical score, a scientific theory, a painting, or a sculpture necessarily begin and end; they have their limits in both time and space. Accordingly, thinking that aspires to be knowledge necessarily requires a reduction. And this leads us to the first notion of quality, because the knowledge will be of one quality or another depending on the method used for this reduction.

A recent paper (Wagensberg 2014b) suggested what the scientific method consists of. Science is maximally objective, intelligible, and dialectic knowledge. Thanks to objectivity, science is maximally universal vis-à-vis both the object (a pear falls just as an apple does) and the subject (the apple falls for any observer at any time and in any place); thanks to intelligibility, science maximally anticipates vis-à-vis time and space (an issue of evident interest for survival); and thanks to its dialectical nature, science necessarily progresses, that is to say, it acquires new knowledge. However, the more complex the content, the shorter the distance you can go with scientific method. We can, then, try another method, that of the communicability of complexities, including those that are unintelligible. This is the method of art. A work of art could be defined in the following way (Wagensberg 2014a, p.27; my translation):

An artwork is a finite piece of reality that distorts an experience of the world to induce in another mind, or in one’s own, an extension of such experience.

More than 30,000 years of history of the human condition vouch for the fact that this method works where science declares itself to be impotent (at least for the moment). A loving passion full of complex nuances cannot perhaps be conveyed scientifically, but it can with a poem, a song, or a simple smile. However, artistic knowledge also has its limitations and for many citizens it is inadequate for conveying some thoughts between mind and mind (though both minds be the same mind). We can, then, turn to a third and final method that will cover any complexity that might still remain without cognitive treatment. This is revealed knowledge, which consists of a single principle:

The revealed method consists of accepting understanding of a complexity of the world simply because an entity that we do not question reveals it to us, be this a deity, consciousness, or simple intuition.

It is clear that quantum physics is a primarily scientific discipline, that painting is a primarily artistic discipline, and that a religion, such as Christianity or Judaism, is a primarily revealed discipline. But none of them is entirely pure as regards their method. There is no physics without an intuition of certain ideas even though scientific method is later applied (the method serves to work on ideas but not to capture them); nor is there any doubt that there can be no art without a certain minimal dose of objectivity and intelligibility; and nor is there a belief that does not seek or use a minimum measure of rationality. Even to mention a miracle perforce involves demonstration that it is not natural. It can perhaps be briefly stated that:

1. Theoretically, there are only three different possible methods for creating knowledge about the reality of the world: the scientific method, the artistic method, and the revealed method.
2. No method is pure in the practice of creating new knowledge but rather a weighted blend of the three different possible methods.

In the terminology of the previous paragraph, real disciplines of human knowledge do not form a division into classes of equivalence (scientific, artistic, or revealed) because no knowledge is purely scientific (without revealed or artistic ingredients), purely artistic (without scientific or revealed components), or purely revealed (without a trace of science or art). This intuition can be explained starting from the fundamental principles that we used to define the scientific method.

Let us consider firstly the principle of objectivity. For this, we start with a drastic and unequal division of the world: me and the rest of reality or, if you prefer, the subject and object of knowledge, or the mind that creates knowledge (the mind capable of observing and understanding) and the slice of reality to be observed and understood. Science, as we understand it today (Wagensberg 2014b), is based on this separation of concepts. At its limit of maximum viability, objectivity occurs when the observation of the world by the mind is achieved without altering in the slightest the observation itself, that is to say, without the observed or the observer being altered by it. In this extreme situation, the principle of objectivity is applied 100 % with all its force. The observation of the movement of a billiard ball does not affect the physical state of the ball nor the state of the onlooker. The extreme opposite of this is the case of a mind attempting to study an object of similar complexity, for example, when it is attempting to understand another mind or when it is attempting to study itself. In this case, it is impossible to prevent the process of observation being affected, though the scientist will always attempt to ensure that the influence is minimal to safeguard to the maximum the degree of universality of the knowledge achieved. We assign a coefficient ß_0_ between zero and one to represent the maximum degree of objectivity achievable in a case of a particular level of complexity. What happens to the scientific shortfall resulting from such a shortfall in objectivity? We can follow a similar line of reasoning when talking about intelligibility. If we accept that the degree of scientific understanding matches the minimum expression of the maximum that is shared, we can also introduce a coefficient ß_i_ to state the degree of compression (and hence also of comprehension) possible in each case. What happens now to the scientific shortfall resulting from such a shortfall in intelligibility? And something similar can be introduced with a possible dialectic coefficient depending on the degree of difficulty we might encounter when guaranteeing the fact that reality can manage to refute our conclusions. We will recognize this dialectic coefficient as ß_d_. What happens to the scientific shortfall overcome by a possible dialectic shortfall?

We can attempt to answer the three questions together because all three stem from the separation of the subject from the object. Let us put ourselves in the situation in which the subject does not attain his maximum objectivity, intelligibility, or dialectic capacity in relation to the object. Let us suppose that the tendency of the mind is to make up for such shortcomings. What can the subject do to make up for that part of the object that infiltrates inseparably into himself? What can the subject do to replace part of the understanding that he finds himself incapable of continuing to compress to its maximum degree? What can the subject do with that understanding that shows itself to be immune to everything that can occur in reality?

There is just one possible answer. The subject must give up the idea that she (the subject) is a necessary and sufficient entity to create objective, intelligible, and dialectic knowledge and accept help from some other class of entity. What should we call that entity capable of taking on the subject’s tasks, in other words, of giving the subject what she needs because her objectivity, intelligibility, and dialectic is not enough for her to attain it by herself? In the first instance, we can call this entity simply *intuition*, that is, the intuition of the subject’s own consciousness. Intuition can be defined as the result of the subject’s experience of the reality of the world, a slight touch between what has already been observed and what has not as yet been observed, a slight friction between what has already been understood and what is not yet understood, a slight graze between the rebuttal already imagined and the rebuttal as yet not imagined. There where the subject cannot go with her objectivity, intelligibility, and dialect, she can with the intuition of her consciousness. The scientific method has made use of another method that we could term the artistic method, a method that only has one principle, which is to address the subject’s intuition when the object becomes so complex that the scientific method reaches its limits. By way of a metaphor, we could define a pair of coefficients between zero and one that we will designate as µ_c_ and µ_a_ respectively for science and art in order to communicate, also metaphorically, the proportions of science and art involved in a particular piece of knowledge.

Just as science eventually reaches its own limits, so too does the artistic approach. How then can the creation of knowledge continue when the intuition of the subject’s own consciousness can do no more? Well, there is a final possibility, and that is for the subject to accept without quibbling the revelation provided by another entity that is not necessarily connected with the subject’s experience but that the subject accepts by definition or as a working hypothesis. We can regard this entity outside the subject as coming from an individual or collective tradition, be it called superstition, conviction, deity, or simply *belief*. Understanding arrived at through belief is accepted as such because of where it comes from. And that’s it. This third method, the revealed method, has no limit, of course. Everything can ultimately be known by this simple method of blind belief. The question of knowing is reduced to a question of will. This third method now covers all the alternatives of any cognitive process; where intelligibility cannot go, intuition can, and where intuition cannot go, belief can. The delicate problem is to intuit when it is still possible to understand, or to believe when it is still possible to intuit. It is as absurd to invent for oneself a god of rain when one already understands the physics of meteorology as it is to attempt to substitute a love poem with a possible psycho-biochemical-mathematical-psychological equation of loving passion.

Pure knowledge does not exist because, among other things, the scientific method is useful for working on ideas but not for capturing them. It is not possible to do science without intuition, nor is it possible to intuit without having, at least temporarily, some belief at the outset. The greatness of science is that it is possible to understand without needing to intuit, and the greatness of art is that it is possible to intuit without needing to understand. The only acceptable priority between science, art, and revelation stems solely from the complexity of the slice of the real world that one is attempting to understand.

In a recent article science is defined as any knowledge obtained following the scientific method, where the latter is suggested to be unique (Wagensberg 2014b). Here are some key concepts redefined in this text: “observation,” “a discipline of knowledge,” and “the comprehension of a slice of reality.” Let us say now, then, that three coefficients between zero and one—µ_c_, µ_a_, µ_r_—weight the degree of science, art, or revelation of any piece of knowledge and that they now represent the so many per one of each of the three possible components.

In order to visualize this idea we can introduce here a logic of fuzzy sets (Zadeh 1965) in the sense that elements do not belong or stop belonging to a particular class but that they belong to all of them, with a particular weight. That is to say, every discipline of knowledge C is the weighted combination of the three alternatives:

4 $$C = \, \mu_{c} C_{c} + \mu_{a} C_{a} + \mu_{r} C_{r} \quad {\text{with}}$$

5 $$\mu_{c} + \, \mu_{a} + \mu_{r} = \, 1\quad {\text{where}}$$

6 $$0 \le \mu_{c} \le 1, \, 0 \le \mu_{a} \le 1, \, 0 \le \mu_{r} \le 1.$$

Accordingly, every discipline of knowledge admits this expression in which the three scientific, artistic, and revealed qualities (c, a, and r) and the three quantities (µ_c_, µ_a_, µ_r_) with which they each contribute all operate. The entropy of knowledge is here a measure of how pure qualities are quantitatively distributed in each real case:

7 $$S = - \mu_{c} \lg_{2} \mu_{c} - \mu_{a} \lg_{2} \mu_{a} - \mu_{r} \lg_{2} \mu_{r} \,{\text{bits}}/{\text{quality}}$$

However, the triple

8 $$\left[ {\mu_{\text{c}} , \, \mu_{\text{a}} , \, \mu_{\text{r}} } \right]$$

gives quite a precise idea of how qualities are distributed. If, for example, one is dealing with political knowledge, a triple (0.2, 0.1, 0.7) would point more to a dictatorship or a theocracy, whereas (0.6, 0.2, 0.2) would be closer to a state of law or a democracy. The same could also be done with the art of Picasso or Borges, which would prove highly scientific in comparison with the art of Van Gogh or Marcel Proust, which would be less scientific though no less great artistically speaking.

Any knowledge about any slice of reality has three defining characteristics: the degree of complexity of its content; the method used to understand this content; and the language in which it is all expressed. The first two, as we have seen, are not entirely independent of each other. When the degree of complexity rises, the method shifts from the scientific to the artistic and from the artistic to the revealed. Language, however, should be added to the triple (8) as a fourth dimension that gives, for example, an idea of the strength of this language, such as through the degree of mathematization that it admits. It can also be represented by a coefficient between zero and one, let us say µ_l_ with l of language (the completely descriptive minimum, the maximum of great mathematical force). In other words:

9 $$\left[ {\mu_{\text{c}} , \, \mu_{\text{a}} , \, \mu_{{{\text{r}},}} \, \mu_{\text{l}} } \right]$$

in which the first three coefficients indicate the type of interdisciplinarity of the methods and the fourth the degree of mathematization of the language.

It is curious to note that in science it is the complexity of the content that draws on the language, whereas in art it is the language that draws on the content. For example, nobody finds it disturbing that Einstein revolutionized physics with the theory of special relativity using classical and familiar mathematical language (although he indeed used new mathematics in order to develop the theory of general relativity). However, no composer working today in the 21st century would dare to present a contemporary musical score written in the language of harmony of Pergolesi.

Comparing quantities is an objective and exacting operation thanks to the relations of order since these define *the greater, equal, or smaller* with total rigor. However, when we are talking about qualities, we do not have something similar with the concepts *better, equal, or worse*. Our treatment of quality does not refer to values of goodness but to values of diversity. It is the quality referred to the idea of the class of equivalence and diversity, not the degree of excellence. For this, one would have to give a scale of values that would necessarily be subjective and difficult to agree on. However, given a piece of knowledge we can now, with the suggested theoretical approach, define four relevant ideas to be borne in mind in any cultural management practice and with which we can begin a discussion. These are described in the final section by way of conclusions.

## Quantity and Quality of Content and Audiences

Consider a piece of knowledge that provides understanding of a slice of reality. In every piece of knowledge it is possible to discern two major concepts: the content (linked to the object of knowledge) and audiences (linked to the subject of knowledge). It is possible to talk of both quantity and quality for each of them.

1. *The quality of the content of knowledge* (*QLC*) can be simply represented by the quadruple defined in (9) or by what we term the Shannon entropy of knowledge (7) qualified by the coefficient of language µ_l_. Greater Shannon entropy means greater interdisciplinarity in this case. Interdisciplinary conversation is appropriate because we are not all ignorant of the same things. The more diverse the content as regards the method used, the greater the potential for this knowledge to create new knowledge, that is to say, the greater its importance. We now undoubtedly come to the intuitive idea that we all have of quality knowledge: that knowledge through which it is possible to generate subsequent knowledge.
2. *The quantity of the content of knowledge* (*QNC*) can, moreover, make reference to the generality of its universality, that is to say, to its degree of universality or even to the size of reality that the piece of knowledge in question represents. It is not easy to give a specific measure here, but we can compare sizes of different contents. For example, the content of the theory of relativity is bigger than Newtonian mechanics because the latter is contained in the former, and Hooke’s law on elasticity is smaller than Newtonian mechanics because the latter contains the former. Thus, the biology of insects is bigger than that of mammals both in the number of individuals and in the number of different species, etc. The larger the size of the content, the higher the probability of its importance for the creation of new knowledge.
  To put it briefly, the quality of a piece of knowledge is assessed by the diversity of the methods used to create it, whereas its quantity is evaluated by the universality of its applicability.
  And we still have two concepts to consider: the quantity and quality of the audience of the content.
3. *The quality of the audience for a piece of knowledge* (*QLA*) is perfectly measured by Shannon entropy (3) if we first define *n* relevant classes of equivalence into which it is appropriate to classify the individuals in the audience of the knowledge in question (readers of a book, spectators at a performance, visitors to a museum, pupils for a course, etc.). It is possible to discuss the criteria in each case for the classes of order (age, educational background, financial status, etc.) or for the classes of equivalence (nationalities, professions, interests, religious beliefs, etc.). Shannon entropy, then, measures (7) the diversity of the audience distributed among these classes. The definition of the classes of equivalence is fundamental here and the ideal criterion in each case must be found.
4. *The quantity of the audience for a piece of knowledge* (*QNA*) is measured using trivial indicators such as the number of consumers (readers, spectators, visitors, etc.). However, these quantities give no idea of the importance of the content to the audience, in other words, they contain no nuance concerning either the part of the audience that has benefited from them or the extent of that benefit. The monument that receives the most visitors anywhere on the planet is undoubtedly the Eiffel Tower in Paris (seven million people visit it each year), yet the visit does not change people’s lives that much. This brings us to a parameter that is difficult to evaluate but which would be much more important when it comes to evaluating both the quantity and the quality of a cultural offering: the change brought about by the consumption of a particular cultural product. For example, and to put it metaphorically, the kilos of conversation caused among the audience.

## Conclusions

For the economy to function, it seems that something has to be growing. The indicators that show everything is going well talk of increases: higher wages, more energy, more transport, more food, more water, more information, more shops, more culture, and so on. That is the logic of quantities, of the little and lots. We compare quantities to assess whether we are shrinking or growing. But how do we compare qualities to determine whether we are doing better or worse? Curiously, the Spanish language is the only one that splits the Latin term *qualitas qualitatis* to make another two: ***cualidad*** (the set of properties and characteristics of things) and ***calidad*** (the result of the comparison of qualities). The first of these can still be quantified. The homogeneous consists of a single *cualidad* (one basket containing ten apples); the heterogeneous of various differently filled *cualidades* (one basket containing two oranges, three apples, and five bananas). There are degrees of heterogeneity, from the minimum homogeneity to the maximum (one basket containing ten different fruits). It is, as we have seen, a question of a magnitude called Shannon entropy (3), which measures the degree of homogeneity of certain preassigned *cualidades*. It is, let us say, the quantity of quality. In fact, what entropy actually measures is not so much the quantity of quality but rather the diversity of quality.

Let us turn to culture. What are we referring to when we say that culture is growing? There is the idea that the best springboard for success is a bit of that selfsame success, though this may not be entirely admissible at the outset. It is somewhat like the whitewashing of success that feeds back into itself to a certain saturation level. This is the undoubted effect of the publication of what we call “best-seller lists.” In the case of non-fiction books, the top places are filled with self-help books that may be very good if they are cookery books but very bad when they deal with other questions to do with the reader’s happiness. A similar trick is commonly used every year when the number of visitors to various museums is calculated and reported. The most usual ploy is to confuse the visitor with a visit. Simply by dividing the museum into two parts (permanent collection and temporary display, for example), each visitor is automatically doubled into two visits. In order words, by virtually dividing the content of the museum by *n*, we trivially multiply its audience by the same factor. Some museums, which cannot bear to go without an annual increase, have managed to accumulate a number of visitors that, if they were real visitors, would only be possible, given the museums’ square footage and their opening times, if these people were to turn up in hordes, piled nine high. Just one number is insufficient to convey cultural merit, and the tyranny of this single number indubitably reduces the quality of any cultural offer.

The issue of how to evaluate each year the quality and quantity of contents and audiences is returned to over and over at almost every international gathering concerning museums. There are many ways of conducting this appraisal, yet none of them is simple. One way is to measure the quantity of conversation generated by the visit and to do so at different characteristic times: (1) conversation between visitors during the visit; (2) conversation during the first family meal after the visit; (3) conversation during the month following the visit… How these evaluations should be done objectively is another issue. It is not easy, but nor is it impossible. Another way is to use an individual’s first visit to arrange two or three telephone interviews to attempt to verify whether the visit has changed any of his habits. Such interviews would be conducted at different times. For example, the first would be held a month after the visit (1), the second six months after (2), and the third a year after (3). The questions asked would be along the lines of: Have you traveled somewhere new? How did you travel? What books have you read since then? Has there been any change in your habits or interests? In short, it is a matter of evaluating how and to what degree an exhibition is capable of affecting the visitor’s cultural activity. In 1993, the *la Caixa* Foundation Science Museum (today known as CosmoCaixa) carried out this kind of interview to evaluate the impact of an exhibition about the Amazon rainforest (Terradas and Wagensberg 2006). We were extremely surprised to find, among other things, that two people had changed the subject of their doctoral theses and another 14 had chosen themes related to those presented in the exhibition. In every case, the change had been brought about by the direct stimulus of the visit.

In general, quantity prevails over quality in every respect. Consequently, cultural creators and administrators may get into the bad habit of only looking for quantity reduced to very banal numbers. As a result, they usually offer stimuli that, in the end, are destined not to seduce on behalf of a certain area of knowledge but to be consumed only as what they are, as stimuli. This occurs when a dinosaur roars or snorts fire in a museum, or when a writer sits down to plot a best-selling novel (i.e., a thousand pages with a secret sect, a prophet and a love interest, an ancient edifice, hidden treasure, corruption and intrigue, etc.). The stimulus is always the first phase in any cognitive process and its purpose is to tip the balance towards *doing* rather than *not doing*. The stimulus is not an end in itself but the starting point that leads to conversation and comprehension. But when the recipient of culture ends up wallowing amid the stimuli, then the cognitive process fails. Intellectual delight reduced to the enjoyment of stimuli can be quite rightly described as pornography.

We mentioned before the unwritten law that states the sum of quantity plus quality is a constant–hence the intuitive strategy of reducing qualities in order to increase quantities. Perhaps this is a monumental misunderstanding. There is another hypothesis to test: quality always ends up dragging quantity after it. Or to put it another way: in the long run, quality ends up having an impact on quantity. We may be surprised to discover that it turns out that this hypothesis is correct. Or to put it another way: that designing with a view to achieving quantity, whatever the impact on quality, is quite simply a huge misunderstanding that we have taken as being right. In short, we all agree that we must put an end to elites, but it is not a matter of doing so by eliminating the members of those elites but by opening the doors so that those on the outside can join them. This is perhaps the only way whereby quantity and quality will not contradict each other but grow harmoniously and in tandem.
